# GDF15 Contributes to the Regulation of the Mechanosensitive Responses of PdL Fibroblasts through the Modulation of IL-37

**DOI:** 10.3390/dj12020039

**Published:** 2024-02-13

**Authors:** Julia Steinmetz, Albert Stemmler, Christoph-Ludwig Hennig, Judit Symmank, Collin Jacobs

**Affiliations:** Department of Orthodontics, University Hospital Jena, 07743 Jena, Germany

**Keywords:** GDF15, IL-37, periodontal ligament fibroblasts, orthodontic tooth movement, tension

## Abstract

During orthodontic tooth movement (OTM), areas of compressive and tensile forces are generated in the periodontal ligament (PdL), a mechanoreactive connective tissue between the teeth and alveolar bone. Mechanically stimulated PdL fibroblasts (PdLFs), the main cell type of PdL, express significantly increased levels of growth differentiation factor 15 (GDF15). In compressed PdL areas, GDF15 plays a fundamental role in modulating relevant OTM processes, including inflammation and osteoclast activation. However, the specific function of this factor in tensile areas has not yet been investigated. Thus, the aim of this study was to investigate the role of GDF15 in the mechanoresponse of human PdLFs (hPdLFs) that were exposed to biaxial tensile forces in vitro. Using siRNA-mediated knockdown experiments, we demonstrated that GDF15 had no impact on the anti-inflammatory force response of elongated hPdLFs. Although the anti-inflammatory markers *IL1RN* and *IL10*, as well as the activation of immune cells remained unaffected, we demonstrated an inhibitory role of GDF15 for the IL-37 expression. By analyzing osteogenic markers, including *ALPL* and *RUNX2*, along with an assessment of alkaline phosphatase activation, we further showed that the regulation of IL-37 by GDF15 modulates the osteogenic differentiation potential of hPdLFs. Despite bone resorption in tensile areas being rather limited, GDF15 was also found to positively modulate osteoclast activation in those areas, potentially by adjusting the IL-37 levels. In light of our new findings, we hypothesize that GDF15 modulates force-induced processes in tissue and bone remodeling through its various intra- and extracellular signaling pathways as well as interaction partners. Potentially acting as a master regulator, the modulation of GDF15 levels may hold relevance for clinical implications.

## 1. Introduction

Orthodontic tooth movement (OTM) plays an important role in the therapeutic correction of tooth malocclusions to ensure their functional properties [1]. Mechanical forces exerted by orthodontic appliances stimulate OTM by activating tissue and bone remodeling processes [2,3,4]. The periodontal ligament (PdL), a connective tissue between the teeth and the alveolar bone, plays a fundamental role in modulating these processes, including tissue inflammation and the activation of bone remodeling cells [5].

Different force zones that are generated in the PdL during orthodontic therapy can be defined [5,6]. On the one hand, the PdL is highly compressed in certain areas, and on the other hand, it is stretched. While compressive forces in particular create a pro-inflammatory microenvironment that promotes bone resorption, tensile forces rather stimulate anti-inflammatory and bone-forming processes [6,7]. In this context, the mechanical stimulation of the PdL, which mainly consists of PdL fibroblasts (PdLFs), triggers certain individual downstream signaling processes in these cells.

As an initial response to compressive and tensile forces, PdLFs modulate the inflammatory response through the expression and secretion of cytokines such as the pro-inflammatory interleukin 6 (IL-6), IL-1β and TNF-α or the anti-inflammatory IL-1RN, IL-10 and IL-37, respectively [6,7,8]. At sites of mechanical compression, these modulate the attraction and activation of further immune cells and the generation of an aseptic transient pro-inflammatory PdL environment [3,9]. Subsequently, activated bone-resorbing osteoclasts promote the resorption of the alveolar bone to enable tooth movement [10,11,12]. In this context, the RANKL/OPG value seems to be particularly relevant for osteoclast activation [13,14]. PdLFs can secrete both receptor activator of NF-kB ligand (RANKL) and osteoprotegerin (OPG) in response to specific stimuli [15]. At compression sides, RANKL/OPG values are increasingly promoting osteoclastogenesis [16,17,18]. RANKL stimulates the differentiation of osteoclast precursors by binding to their transmembrane receptor RANK, which is blocked by OPG acting as a decoy receptor for RANKL [19]. In elongated PdL areas, the attraction and activation of osteoclasts are limited due to increasing OPG levels [20]. However, to ensure the stability of the new tooth position, bone formation is particularly stimulated on the sides of tensile forces by activating osteoblast differentiation [21]. Remarkably, PdLFs possess a capacity to differentiate into functional osteoblasts and thus promote bone formation [22]. Increased expressions of osteogenic markers such as alkaline phosphatase (ALP, gene *ALPL*), runt-related transcription factor 2 (RUNX2), osteocalcin (OCN) and osteopontin (OSP) as well as increased ALP activity and the formation of calcium deposition were detected [23,24,25,26].

Our previous studies have highlighted the role of growth differentiation factor 15 (GDF15) in the mechanoresponse of human PdLFs (hPdLFs) [25,27,28], supported by the results of Li et al. [29]. GDF15 is known as a member of the transforming growth factor (TGF)-β and bone morphogenetic protein (BMP) family [30]. It is mainly produced in pathogenic situations and during cellular stress and is therefore associated with many diseases, such as cancer, obesity, insulin resistance, type 2 diabetes and cardiovascular diseases [31]. Aging is also associated with increased GDF15 serum levels [32]. However, under physiological conditions it is only weakly produced in most tissues [31].

Synthesized as precursor protein, the disulfide-linked dimerization of GDF15 occurs prior to secretion [33]. Extracellular GDF15 can bind to cell type-specific, membrane-bound receptors, with the glial cell line-derived neurotrophic factor family receptor α-like (GFRAL) and the activin receptor-like kinases (ALKs) being the best characterized [34,35,36]. In this context, GDF15 has been reported to stimulate the expression of the osteogenic markers in bone marrow-derived precursors, potentially promoting their differentiation [27]. Moreover, we recently reported that hPdLFs themselves also express potential GDF15 receptors and that increased expression levels for *ALPL* and *RUNX2* as well as increased ALP activation could be detected upon prolonged stimulation [25]. In addition to its receptor-mediated signaling, intracellular GDF15 has also been reported to exert specific regulatory functions [37,38].

During mechanical force exposure, the GDF15 expression is significantly increased in hPdLFs, in compressed as well as in stretched cells [25,27,28,29]. In compressed areas of the PdL it acts pro-inflammatory and promotes the activation of osteoclasts [28]. However, the functions of GDF15 in the mechanoresponse of PdL fibroblasts to tensile forces have not been investigated so far. The aim of this study was therefore to examine the impact of GDF15 on the response of hPdLFs to biaxial tensile forces with regard to their osteogenic differentiation and their modulation of inflammatory processes and bone-resorbing cells.

## 2. Materials and Methods

### 2.1. Cell Culture

Human periodontal ligament fibroblasts (hPdLFs, Lonza, Basel, Switzerland) were grown in DMEM with 4.5 g/L glucose (Capricorn Scientific GmbH, Ebsdorfergrund, Germany) with 10% fetal bovine serum (FBS; Thermo Fisher Scientific, Carlsbad, CA, USA), 1% penicillin/streptomycin (Gibco, Thermo Fisher Scientific, Carlsbad, CA, USA) and 1% L-ascorbid acid (Sigma Aldrich, St. Louis, MO, USA). Cells were regularly subcultured when a confluency of 75% was reached. Subcultures four to eight were used.

THP1 monocytic cells (DSMZ, Braunschweig, Germany) were cultured in RPMI 1640 with L-glutamine and sodium bicarbonate (Capricorn Scientific GmbH, Ebsdorfergrund, Germany) supplemented with 10% FBS and 1% penicillin/streptomycin.

### 2.2. Tensile Strain Application

For experiments, 10^5^ hPdLFs were seeded per well on flexible bottomed 6-well cell culture plates (BioFlex^®^ Culture Plates, FLEXCELL^®^, Asbach, Germany) coated with pronectin. When reaching a confluence of 75%, cells were strained with a tensile force of 15.9% for 12 h according to Nazet et al. [39]. Therefore, spherical cap silicone stamps (radius 22 mm, high 7.1 mm) made of S4 suhy dental a-silicone (Bisico, Bielefeld, Germany) were clamped onto the bottom of the plates. The control was left unstrained.

### 2.3. siRNA-Mediated Down-regulations of GDF15 and IL37

Down-regulations of *GDF15* and *IL37* were performed as previously described [28]. Briefly, 50 nM small interfering RNA targeting human mRNA sequences (*GDF15* siR: Santa Cruz Biotechnology, Inc. Dallas, Taxas, USA; *IL37* siR: Thermo Fisher Scientific, Carlsbad, CA, USA) were transfected into cells using a Lipofectamine^TM^ 2000 (Thermo Fisher Scientific, Carlsbad, CA, USA) in OptiMem I-reduced serum medium (Thermo Fisher Scientific, Carlsbad, CA, USA) containing 1% penicillin/streptomycin. As control (ctrl siR), BLOCK-iT Alexa Fluor red control siRNA (Thermo Fisher Scientific, Carlsbad, CA, USA) was used. After a 5 h transfection, the reagent was replaced by the fibroblast culture medium.

### 2.4. RNA Extraction and Quantitative Polymerase Chain Reaction (PCR)

RNA isolation was performed as previously described byTRIzol Reagent (Thermo Fisher Scientific, Carlsbad, CA, USA)/1-bromo-3-chloropropane RNA isolation and purification with RNA Clean & Concentrator-5 kit (Zymo Research, Freiburg, Germany) [40]. Subsequently, the RNA quality and quantity were checked with a Nanodrop 2000 (Avantor, Radnor, PA, USA). SuperScript™ IV Reverse Transcriptase and Oligo(dt)_18_ primers (both Thermo Fisher Scientific, Carlsbad, CA, USA) were used for cDNA synthesis. Quantitative expression analysis by PCR was performed with Luminaris Color HiGreen qPCR Master Mix (Thermo Fisher Scientific, Carlsbad, CA, USA) with the qTower3 (Analytik Jena, Jena, Germany). The sequences of the used primers are displayed in Table 1 with ribosomal protein L22 (*RPL22*) and TATA box binding protein (*TBP*) as reference genes. The primer quality and specificity were checked via melting curve analysis and agarose gel electrophoresis. The efficiency correction ΔΔCT method was used for data analysis.

### 2.5. Enzyme-Linked Immunosorbent Assay (ELISA)

To quantify the IL-37 secretion, cell culture supernatants were analyzed via ELISA (Abcam, Cambridge, UK, IL-37 ab 213798) according to the manufacturer’s protocols.

### 2.6. THP1 Activation Assay

To visualize the inflammatory response of hPdLFs, the activation of THP1 monocytic cells was analyzed as previously described [40]. CMFDA (Thermo Fisher Scientific, Carlsbad, CA, USA)-stained THP1 cells were added to each well of stimulated hPdLFs. After the removal of non-adherent cells, the remaining adherent cells were fixed for 10 min in 4% paraformaldehyde (PFA). DAPI (1:10,000 in PBS) staining was used to visualize cell nuclei. Membrane cut-outs were embedded with Mowiol^®^ 4-88 (Carl Roth, Karlsruhe, Germany) on glass object slides.

### 2.7. Osteoclast Activation Assay and TRAP Staining

To visualize the osteoclast activation by hPdLFs, the supernatant of stimulated fibroblasts were isolated and used for six-day stimulation with phorbol 12-myristate 13-acetat (PMA, 100 ng/mL, 2 days)-prestimulated THP1 macrophage-like cells as previously described [25]. Subsequently, cells were fixed in 4% PFA for 10 min and in 50:50 acetone/ethanol for 1 min, air-dried and stained for tartrate-resistant acid phosphatase (TRAP) as previously described [25]. To identify multinucleated osteoclasts, SYTO nucleic acid staining (Thermo Fisher Scientific, Carlsbad, CA, USA) was performed.

### 2.8. ALP Activity Assay

To visualize the alkaline phosphatase (ALP) activity, NBT/BCIP staining (Thermo Fisher Scientific, Carlsbad, CA, USA) was performed as previously described [25].

### 2.9. Immunofluorescent Staining

To detect intracellular IL-37 levels, immunofluorescent staining was performed as previously described [41,42]. Briefly, stimulated hPdLFs were first fixed with 4% PFA for 10 min and washed with 1x PBS/0.1% Triton X (Sigma Aldrich, St. Louis, MO, USA). Bovine serum albumin (BSA, 4%, Seqens IVD, Limoges, France) in washing buffer was used to block unspecific antibody binding sites. The primary antibody rabbit-anti IL-37 (Abcam, Cambridge, UK, IL-37 ab278499, 1:100 in blocking solution) was applied for 3 h. After further washing steps, the secondary antibody goat-anti-rabbit Cy5 (111-175-144; Jackson Immuno Research, West Grove, PA, USA; 1:1000 in blocking solution) was applied for 45 min. After washing, 10 min nucleic staining with DAPI was performed. Membrane cut-outs were embedded in Mowiol^®^ 4-88 (Carl Roth, Karlsruhe, Germany) on glass slides.

### 2.10. Microscopy and Image Analysis

Imaging for the evaluation of THP1 activation and immunofluorescent staining was performed with the inverted confocal laser scanning microscope TCS SP5 (Leica, Wetzlar, Germany). The NBT/BCIP and TRAP stainings were imaged with a Primovert microscope (Carl Zeiss Company, Oberkochen, Germany). To analyze the number of THP1 cells and the intensity of the IL-37 immunostaining, Fiji software (https://imagej.net/Fiji; version number 1.52p, accessed on 10 January 2021) was used. Mean grey values (MGVs) were measured for IL-37 staining in the cytoplasm of 180 cells per condition, including background correction as previously described [41,42]. Microscopic imaging of each experiment was performed under identical settings for each treatment condition. To avoid overexposure, settings were limited to the most intense condition. The intensity of the MGVs was visualized as a thermal LUT, displayed as values normalized to control conditions and presented as percentage changes. For analyzing osteoclast activation, mutltinucleated TRAP-positive cells were counted by overlaying the TRAP and SYTO nucleic acid stainings for each image.

### 2.11. Statistics

Statistical analyses were performed with Graph Pad Prism 9 (https://www.graphpad.com; version number: 10.1.2, accessed on 26 November 2021). All experiments were performed in technical duplicates and independently repeated at least three times. One-way ANOVA and a post hoc test (Tukey) were used as statistical tests. Significance levels: */# *p*-value < 0.05, **/## *p*-value < 0.01, ***/### *p*-value < 0.001.

## 3. Results

### 3.1. GDF15 Limits IL-37 in Stretched PdL Fibroblasts without Impacting Their Anti-Inflammatory Mechanoresponse

**Figure 1:**
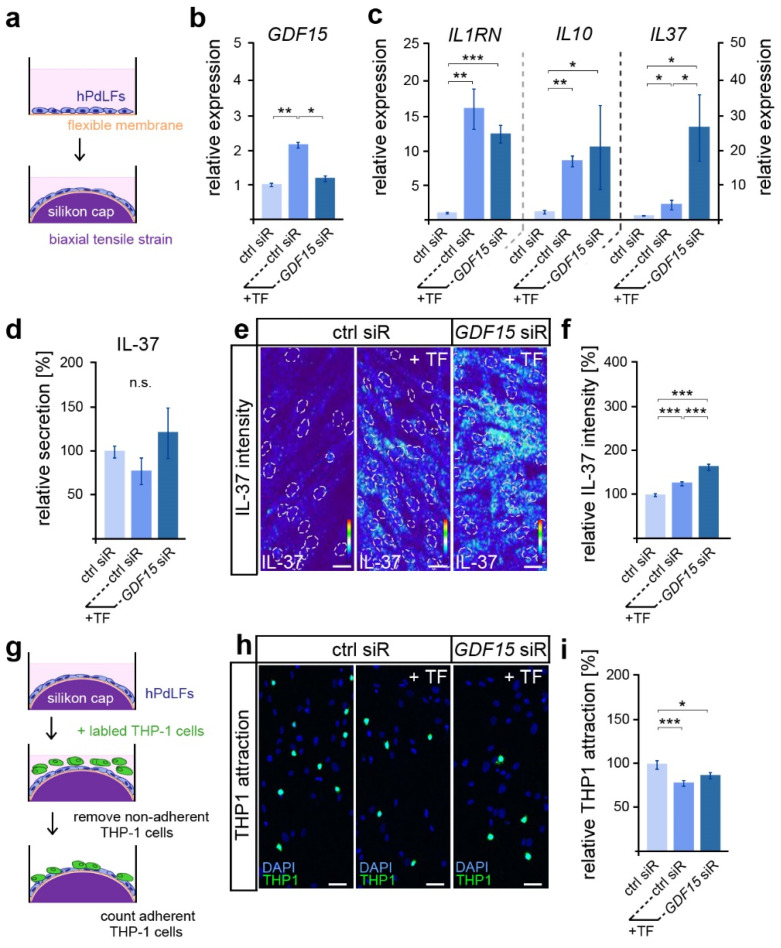
GDF15 limits IL-37 in elongated hPdLFs without impacting their anti-inflammatory mechanoresponse. (**a**) Experimental model showing human periodontal ligament fibroblasts (hPdLFs) cultured on flexible membranes and stressed with a biaxial tensile forces (+TF) of 15.9% for 12 h by silicone stamps. (**b**,**c**) Relative expression analysis of *GDF15* (**b**) and genes encoding the anti-inflammatory cytokines IL-1RN, IL-10 and IL-37 (**c**) in elongated hPdLFs treated with *GDF15* siRNA (*GDF15* siR) compared to unstressed controls (ctrl siR). (**d**) Secretion analysis of IL-37 in the supernatant of stimulated hPdLFs in relation to the unstimulated control. (**e**,**f**) Representative micrographs of the IL-37 immunofluorescent intensities of stimulated hPdLFs (**e**) analyzed in relation to the unstimulated control (**f**). IL-37 staining intensities are shown as thermal LUTs, and cell nuclei are visualized by dotted circles. (**g**) Experimental model showing the activation of THP1 monocytic cells by stimulated hPdLFs to visualize their inflammatory response. (**h**,**i**) THP1 activation assay showing adherent Alexa488-labled THP1 monocytic cells (green) on mechanically stressed *GDF15*-deficient hPdLF (blue, DAPI; **h**) displayed in relation to the unstimulated control (**i**). * *p* < 0.05; ** *p* < 0.01; *** *p* < 0.001; n.s., not significant; One-way ANOVA and post hoc test (Tukey). Scale bars: 20 μm.

Given the important functions of GDF15 in modulating the inflammatory mechanoresponse of hPdLFs to compressive stress [28], we were interested in its possible involvement in the stress response to biaxiale tensile forces (Figure 1a). As described previously [27], a significantly increased *GDF15* expression was observed after 12 h of tensile stress via quantitative PCR (Figure 1b). Using the siRNA-guided down-regulation of *GDF15* in stretched hPdLFs (see Figure 1b), we further investigated its role in the tensile stress-mediated increase in the expression of genes coding for the anti-inflammatory markers IL-1RN, IL-10 and IL-37 (Figure 1c). Thereby, *GDF15* deficiency significantly increased the *IL37* levels in stretched hPdLFs, but had no impact on the other markers.

Since IL-37 is well characterized for its anti-inflammatory activity [43], we further assessed IL-37 secretion via ELISA (Figure 1d). However, no significant differences in the secretion levels were detected, neither by tensile stress nor by *GDF15* knockdown. As IL-37 can also act through intracellular pathways [43], we next used quantitative immunofluorescence to analyze intracellular expression levels (Figure 1e,f). Overall, the IL-37 levels were significantly increased in stretched hPdLFs. The knockdown of *GDF15* further promoted the mechano-related increase in IL-37, suggesting a limiting role of GDF15 in IL-37 signaling under stress conditions.

To investigate the inflammatory level in elongated hPdLFs, we evaluated the activation of monocytic THP1 cells (Figure 1g). They can differentiate into adherent macrophages after the detection of pro-inflammatory signals, and thus visualize the inflammatory response of hPdLFs. Compared to the basic activation level of immune cells, which is always observed in cultured hPdLFs, biaxial tensile forces induced a significantly lower activation of THP1 cells (Figure 1h,i). This indicates enhanced anti-inflammatory signals through the stretched hPdLFs. However, *GDF15* deficiency had no impact on THP1 activation in elongated hPdLFs. Thus, our data suggest that although GDF15 exerts an inhibitory influence on the expression of the anti-inflammatory IL-37 in hPdLFs, it does not affect the overall anti-inflammatory response to tensile forces.

### 3.2. GDF15 Promotes the Differentiation of Stretched PdL Fibroblasts

Given the multiple influences of GDF15 on bone remodeling [44], we investigated the extent to which the factor is also relevant to the osteogenic processes in hPdLFs, which can be induced by tensile forces [21].

**Figure 2:**
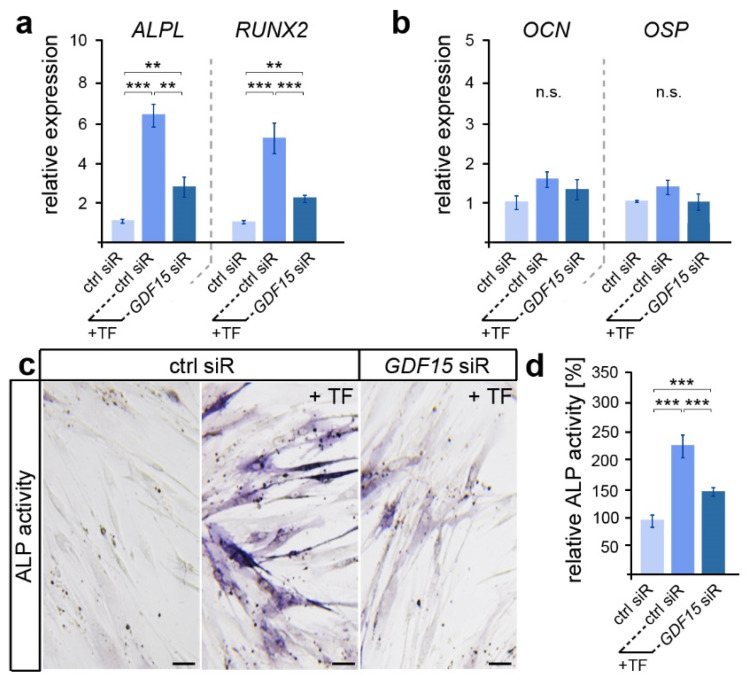
GDF15 promotes the osteogenic differentiation of elongated hPdLFs. (**a**,**b**) Relative expression analysis of genes encoding the early osteogenic markers ALP (gene: *ALPL*) and RUNX2 (**a**) and the late osteogenic markers OCN and OSP (**b**) in 12 h tensile force-stressed (+TF) hPdLFs treated with *GDF15* siRNA (*GDF15* siR) compared to the unstressed control (ctrl siR). (**c**,**d**) Representative microphotographs of the alkaline phosphatase activity (dark blue; ALP activity, **c**) analyzed in relation to the unstressed control (**d**). ** *p* < 0.01; *** *p* < 0.001; n.s., not significant; One-way ANOVA and post hoc test (Tukey). Scale bars: 25 μm.

Here, we examined the expression of genes encoding the osteogenic markers ALP (gene *ALPL*), RUNX2, OCN and OSP (Figure 2a,b). While early expressed osteogenic genes such as *ALPL* and *RUNX2* showed increased levels after the application of biaxial tensile forces, no significant differences in the expression of the late osteogenic marker *OCN* and *OSP* were detected. However, *ALPL* and *RUNX2* levels were significantly reduced in stretched hPdLFs with a diminished *GDF15* expression. A subsequent analysis of alkaline phosphatase (ALP) activity confirmed the supporting role of GDF15 in the osteogenic differentiation of elongated hPdLFs (Figure 2b,c), as GDF15-deficient stretched hPdLFs also showed reduced ALP activity.

**Figure 3:**
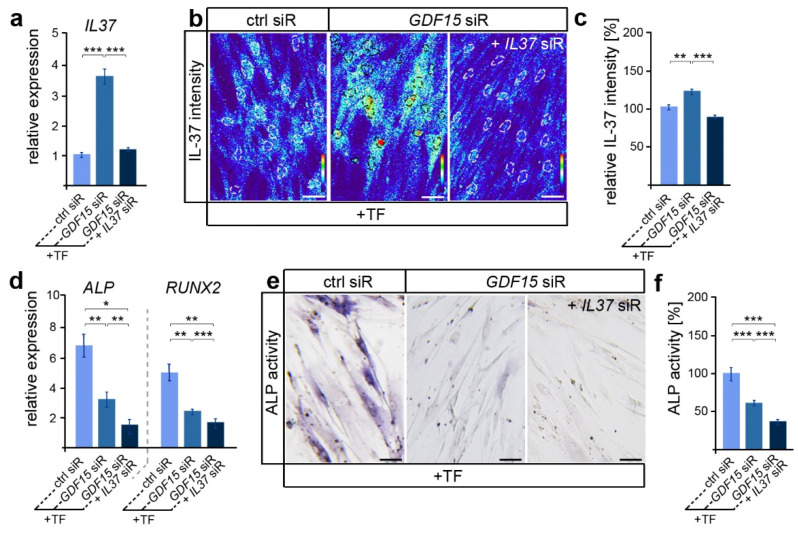
By limiting IL-37, GDF15 adapts the osteogenic differentiation potential. (**a**) Relative expression analysis of IL-37 in tensile force-stressed (+TF) hPdLFs treated with *GDF15* siRNA (*GDF15* siR) only or in combination with *IL37* siR (*GDF15* siR + *IL37* siR) compared to stretched controls (ctrl siR). (**b**,**c**) Representative micrographs of the IL-37 immunofluorescent intensities of stimulated hPdLFs (**b**) analyzed in relation to the elongated control (**c**). IL-37 staining intensities are shown as thermal LUTs, and cell nuclei are visualized by dotted circles. (**d**) Relative expression analysis of genes encoding ALP (gene: *ALPL*) and RUNX2. (**e**,**f**) Representative microphotographs of the alkaline phosphatase activity (dark blue; ALP activity, **f**) analyzed in relation to the elongated control (**f**). * *p* < 0.05; ** *p* < 0.01; *** *p* < 0.001; One-way ANOVA and post hoc test (Tukey). Scale bars: 25 μm.

In view of the participation of IL-37 in the regulation of osteoblastogenesis in various cell types [43], we were now interested in whether GDF15 might contribute to the differentiation of elongated hPdLFs through the modulation of IL-37 signaling. Thus, we reduced the *IL37* expression levels via siRNA-mediated knockdown in *GDF15*-deficient hPdLFs resulting in levels comparable to those of the stretched control (Figure 3a). Further, increased IL-37 protein levels in *GDF15*-deficient hPdLFs were balanced to the control level due to the *IL37* siRNA treatment (Figure 3b,c). Interestingly, *GDF15-* and *IL37*-double-deficient hPdLFs showed further reductions in *ALPL* and *RUNX2* expression levels (Figure 3d) as well as ALP activity (Figure 3e,f), emphasizing a positive impact of IL-37 on their osteogenic differentiation. This increased expression of IL-37 does not correlate with the reduced osteogenic differentiation observed in the *GDF15* single knockdown fibroblasts (see Figure 2 and Figure 3a–c), emphasizing another function of IL-37 in elongated hPdLFs, which might be limited by GDF15.

### 3.3. GDF15 Balances the Activity of Osteoclasts in Elongated PdL Fibroblasts Potentially through an IL-37-Dependent Pathway

**Figure 4:**
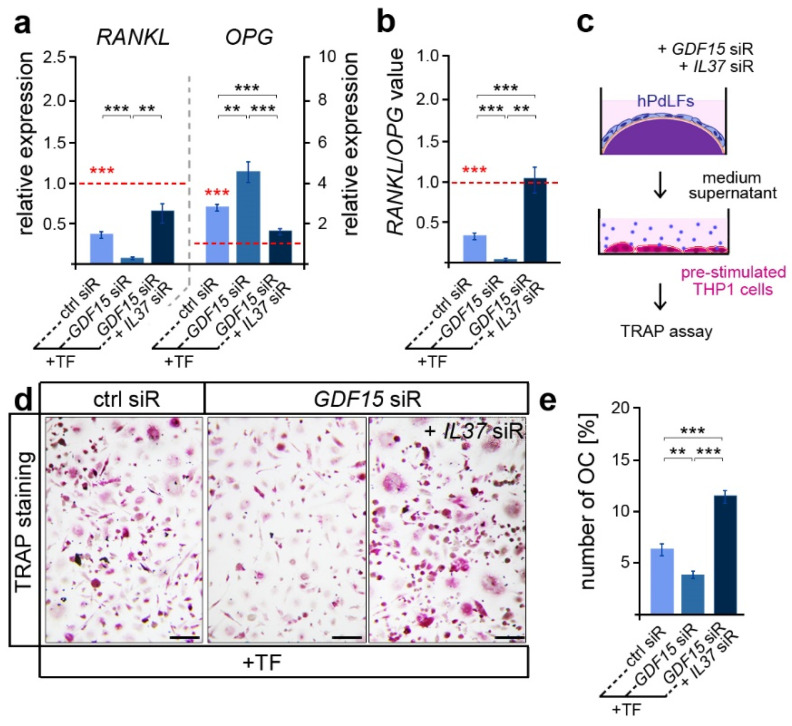
GDF15 balances osteoclast activation potentially by modulating IL-37 signaling. (**a**) Relative expression analysis of osteoclastogenic regulators *RANKL* and *OPG* in tensile force-stressed (+TF) hPdLFs treated with *GDF15* siRNA (*GDF15* siR) or combined with *IL37* siR (*GDF15* siR + *IL37* siR) compared to unstressed controls (ctrl siR, red dotted line; red significances). (**b**) *RANKL*/*OPG* values of expression data in relation to the unstimulated control (red dotted line, red significances). (**c**) Experimental model indicating osteoclast activation assay with pre-stimulated THP1 cells. (**d**,**e**) TRAP assay of pre-stimulated THP1 cells cultured for 6 days in the medium supernatant of stressed hPdLFs analyzed in (**e**) as percentages of the multinucleated osteoclasts per cell. ** *p* < 0.01; *** *p* < 0.001; One-way ANOVA and post hoc test (Tukey). Scale bars: 25 μm.

Besides its important role in promoting osteoblast differentiation, IL-37 was also reported to inhibit osteoclastogenesis and bone resorption [45]. Thus, we further focused on osteoclast activation, which is typically limited in tensile areas of the PdL [20]. In line with this, we detected reduced *RANKL* and increased *OPG* expression levels in elongated hPdLFs compared to those of the unforced controls (Figure 4a, red dotted line and significance), which resulted in lower *RANKL*/*OPG* values (Figure 4b red dotted line and significance). *GDF15* deficiency significantly decreased the *RANKL*/*OPG* levels, which were based on a reduced *RANKL* and increased *OPG* expression (Figure 4a,b; black significances). Interestingly, this might indicate a promoting role of GDF15 on alveolar bone resorption. However, by further limiting IL-37 over-expression in *GDF15*-deficient hPdLFs through the *IL37* siR treatment, *RANKL*/*OPG* values were significantly up-regulated based on increased *RANKL* and reduced *OPG* levels. In line with the literature, this clearly indicates the inhibitory function of IL-37 on osteoclastogenesis.

In order to test corresponding assumptions based on *RANKL*/*OPG* values, we examined the activation of osteoclasts by respective hPdLFs. To this end, pre-stimulated THP1 macrophages were cultured with the medium supernatant of hPdLFs for six days to stimulate osteoclastogenesis (Figure 4c). A TRAP assay revealed a reduced number of multinucleated osteoclasts when stimulated with the supernatant of *GDF15*-deficient elongated hPdLFs (Figure 4d,e), indeed indicating a promoting role of this cytokine. The extent to which this reduction in OC activation is related to the excessive IL-37 expression in *GDF15*-deficient fibroblasts was determined through an additional siRNA-mediated reduction in IL-37 levels. In line with the increased *RANKL*/*OPG* values, the number of multinucleated OCs was enhanced when they were stimulated with the supernatant of elongated double knockdown hPdLFs.

In summary, our results imply a multifunctional and complex role of GDF15 in elongated PdL fibroblasts. While on the one hand, it appears to promote the osteogenic differentiation of those cells, it also seems to have a stimulating role in osteoclast activation. Furthermore, our data show a critical role of GDF15/IL-37 signaling in these processes.

## 4. Discussion

During orthodontic tooth movement, the precise control of inflammatory processes and the remodeling of the alveolar bone are of major relevance to the orthodontic therapy duration and outcome. However, these processes depend on patient- and treatment-specific conditions and involve risks [46]. These include periodontal inflammation and attachment or even tooth loss. Understanding the underlying mechanobiological processes triggered by compressive and tensile forces as well as the potentially identifying key factors might help to improve future orthodontic treatment concepts enabling patient-specific therapy.

In this context, GDF15 appears to be a promising target for the modulation of tooth movement due to its pro-inflammatory role in the mechanical response of the periodontal ligament cells to compressive forces [28]. Nevertheless, the precise role of GDF15 in the tensile zones of the PdL are not yet known, although increased expression and secretion have already been detected [27]. We have now shown that upon application of tensile forces, GDF15 appears to be particularly relevant for the osteogenic differentiation of local PdL fibroblasts, rather than for their anti-inflammatory response. However, we also identified a pro-osteoclastogenic role of GDF15 in elongating fibroblasts, which seems to be interesting in combination with its pro-osteogenic function. In this context, we have further identified IL-37 as a potential downstream target of GDF15, being an important key player in modulating bone-remodeling cells.

The precise role of GDF15 in modulating inflammatory processes seems to be highly dependent on the cellular context as well as on the specific stress stimuli [47,48]. Its anti-inflammatory and tissue-protective effects have been described in several studies by directly influencing the functionality of immune cells such as macrophages, neutrophils, dendritic cells, natural killer cells and T lymphocytes [48]. However, the impact of GDF15 is not limited to immune cells. For instance, GDF15 promotes the activation and differentiation of lung fibroblasts, particularly contributing to the progression of idiopathic pulmonary fibrosis [49]. In this respect, receptor-mediated differences appear to be particularly relevant to the effect of GDF15 due to the potential cell type-specific expression of different receptors [33]. PdL fibroblasts express membrane receptors of the ALK family, in particular ALK1, ALK2 and ALK5 [25], which have been reported to exert GDF15-mediated signaling in various cell types [35,36,50]. However, the distinct anti-inflammatory response of these cells to tensile forces appears to be independent of GDF15 as siRNA-mediated silencing had no effect on the expression of the classical markers *IL10* and *IL1RN* or on the activation of monocytic immune cells. This seems to be in line with other studies also showing no direct influence of GDF15, at least on IL-10 [51,52]. Interestingly, prolonged exposure to recombinant human GDF15 (rhGDF15) significantly enhanced the *IL1RN* expression in PdLFs, suggesting a more complex signaling cascade that also appears to involve changes in cell fate [25]. In addition to IL-10 and IL-1RN, IL-37 is also a cytokine with significant anti-inflammatory properties [45], and its expression can be modulated by tensile forces in PdL cells [7]. Although we did not detect GDF15-dependent changes in the anti-inflammatory force response, the RNA and protein levels of IL-37 were affected by GDF15 signaling in human PdLFs. That raises the question of what effects this might have if not altering the inflammatory response.

In addition to the inflammatory mechanoresponse, tensile forces specifically stimulate the activation and differentiation of osteoblasts, which ensure the stability of teeth within the alveolar bone by forming new bone material [21]. Members of the TGF-ß/BMP-family are well known for their potential to modulate osteoblast differentiation [44,53]. Even though Westhrin et al. reported an anti-osteogenic effect of GDF15 on human bone marrow (BM)-derived mesenchymal stem cells (BMSCs), GDF15 appears pro-osteogenic in several other studies. In line with our own study on primary osteoblasts [27], 7 day stimulation with rGDF15 significantly increased the number of BM-derived mesenchymal stromal cells and the expression of the pro-osteogenic markers RUNX2 and Osterix (OSX) [54]. Furthermore, enhanced osteoblast differentiation was also reported in prostate cancer cells when transfected with *GDF15* cDNA [55]. Furthermore, elevated levels of GDF15 associated with prostate cancer facilitated bone metastasis and bone turnover, while the deletion of GDF15 in this context inhibited osteoblast differentiation and mineralization [56]. Most recently, we reported that prolonged exposure to rhGDF15 stimulates the *ALPL* and *RUNX2* expressions, as well as increases ALP activity and the formation of calcified nodules in PdL fibroblasts [25]. Here, we could now demonstrate that GDF15 also exerts a promoting role in tension-induced osteoblastogenesis as the *ALPL* and *RUNX2* expressions as well as ALP activity were reduced in elongated *GDF15*-deficient fibroblasts. Due to the rapid changes in the osteogenic differentiation of hPdLFs after siRNA transfection within 12 h, we speculate that the intracellular functions of GDF15 are rather relevant to this effect. In this context, nuclear GDF15 has been reported to influence TGF-β1-induced signaling pathways of the suppressors of mothers against decapentaplegic (SMAD) proteins [37], which are crucial in osteoblast differentiation [57]. Min et al. [37] reported that GDF15 restricts the formation of DNA/SMAD complexes, although neither by directly binding to DNA nor to SMAD proteins. However, IL-37 has been shown to directly bind to SMAD3 and translocate into the nucleus when phosphorylated to modulate gene expression [58,59]. Since we have now demonstrated a limiting effect of GDF15 on the IL-37 expression, this might be a potential pathway by which GDF15 indirectly interferes with SMAD signaling. However, other signaling pathways could also be affected, as has been shown for GDF15 and the stress response of osteoblast-like cells [60,61]. Based on our data, we speculate that GDF15 modulates the level of osteoblastogenesis at least in part by limiting IL-37, as a reduction in an excessive IL-37 expression in stretched GDF15-deficient cells has an additional osteogenic effect. Regarding IL-37, this appears to be consistent with the study by Ye et al. in which an increased differentiation of BMSCs into osteoblasts was demonstrated when stimulated extracellularly with IL-37 [62]. In contrast to this study, however, we observed only changes in the intracellular IL-37 concentration, while values in the extracellular environment remained unaffected. Therefore, we speculate that IL-37 may stimulate osteoblast formation not only through membrane receptor activation but also through direct intracellular interactions. Regarding the limiting role of GDF15 in IL-37 expression and thus in osteoblastogenesis, this initially seems contradictory given the predominant processes on the tensile side. However, we hypothesize that GDF15 might be important for the balance in bone formation by modulating facilitating and restricting factors.

This also appears to be relevant for the activation of osteoclasts on the tensile side. This process is normally limited by the increased secretion of OPG after the application of tensile forces [20]. We also detected an increased *OPG* expression, and GDF15 appeared as a pro-osteoclastogenic factor in elongated PdL fibroblasts as the knockdown resulted in reduced *RANKL*/*OPG* values. However, we did not investigate secretion and therefore cannot rule out differences at the protein level. Nevertheless, Li et al. reported comparable results in compressed PdL cells, where the siRNA-mediated down-regulation of GDF15 decreased their RANKL/OPG levels [29]. In this context, we have recently shown that compressed PdL fibroblasts increasingly activate osteoclast differentiation when previously stimulated with rhGDF15 for 36 days [25]. Our results are thus consistent with several other studies on various cells with osteoclastogenic differentiation potential, where stimulation with GDF15 also led to an increased maturation of this cell fate [29,63,64]. In contrast, Vanhara et al. reported that GDF15 inhibits osteoclastogenesis in RAW264.7 macrophages and bone marrow mononuclear progenitor cells in a dose-dependent manner [65]. These divergent results should be viewed critically, as particular stimulation conditions may differ between studies. In addition, it was also revealed that many products of recombinant GDF15 proteins might be contaminated with TGF-β1 [66], which also activates osteoclast differentiation [53]. However, based on our experimental approach, we hypothesize intracellular effects of GDF15 in modulating the *OPG*/*RANKL* expression through PdL fibroblasts rather than direct effects of this factor on osteoclast precursors. Nevertheless, we cannot rule out direct effects, which could be addressed, for example, by adding GDF15-blocking antibodies to the medium supernatant prior to the stimulation of osteoclast precursors. The exact mechanism is beyond the scope of this study and needs to be addressed in more detail in the future.

Regarding IL-37, this multifunctional regulator has been reported not only to have direct effects on osteoclast precursors [67,68], but also to inhibit the expression of inflammatory cytokines related to osteoclast differentiation, such as RANKL [69]. Similarly, we found increased levels of RANKL when overexpressed IL-37 was down-regulated in prolonged *GDF15*-deficient PdL fibroblasts. Thus, the modulating function of GDF15 for IL-37 signaling may preserve the potential of PdL cells to activate bone-resorbing cells when required. This seems particularly clinically relevant as the intensity and correct localization of tensile and compressive zones may change during orthodontic treatment [70]. In vitro, therefore, it is clearly a simplified theoretical model with separate force zones. However, orthodontic therapeutic procedures can also lead to tooth tipping resulting in tensile and compressive zones on both sides of the tooth in immediate proximity [71]. This requires precise and adaptable regulation in bone formation and resorption processes. In this context, our data indicate that GDF15 may contribute to this complex regulation, in part by modulating IL-37 signaling.

Clinically, modulating GDF15 levels appears to be particularly interesting for the adjustment of the mechanoresponse of PdL fibroblasts, addressing the modulation of the inflammatory responses and activation of bone-remodeling cells. In this context, the use of blocking antibodies targeting GDF15 is currently the most promising option [72]. This also seems to be important in light of the increased serum GDF15 levels in a variety of diseases, which may have a potential impact on the mechanoresponse of PdL fibroblasts [47]. However, further in vivo studies will be required to clarify the precise role of GDF15 in the orthodontic context.

## 5. Conclusions

The present study provides new details on the role of the multifunctional regulator GDF15 in the mechanobiology of periodontal ligament cells. In response to tensile forces, increased GDF15 levels promote the osteogenic differentiation and modulates the activity in promoting osteoclast differentiation. Therefore, limiting IL-37 signaling using GDF15 appears to be particularly relevant for the tension-induced changes in the osteogenic properties of PdL fibroblasts and osteoclast activation. Since GDF15 also modulates corresponding processes in response to compressive forces, we hypothesize that GDF15 may act as a master regulator through its multiple intra- and extracellular mechanisms by interacting with various downstream modulators that may differ on both sides. Considering the future possibilities of clinically modulating GDF15 levels, new approaches for patient-specific orthodontic treatment might be an interesting objective.

## Figures and Tables

**Table 1:** qPCR primer sequences of all analyzed genes with gene symbol, NCBI gene ID and primer sequence in the 5′-3′ direction with fw as forward and rev as reverse.

Gene	Gene Symbol	NCBI Gene ID	Primer Sequence
Alkaline phosphatase	*ALPL*	249	fw ACTGCAGACATTCTCAAA rev GAGTGAGTGAGTGAGCA
Bone gamma- carboxyglutamate protein	*BGLAP* (alias *OCN*)	632	fw GCAGCGAGGTAGTGAAGAGA rev AGCAGAGCGACACCCTAGA
Growth differentiation factor 15	*GDF15*	9518	fw CCGAAGACTCCAGATTCCGA rev CCCGAGAGATACGCAGGTG
Interleukin 10	*IL10*	3586	fw AGCCATGAGTGAGTTTGACA rev AGAGCCCCAGATCCGATTTT
Interleukin 1 receptor antagonist	*IL1RN*	3557	fw GATGTGCCTGTCCTGTGTCA rev ACTCAAAACTGGTGGTGGGG
Interleukin 37	*IL37*	27178	fw AGTCCGATTCTCCTGGGG rev TTTATAGGGCTCAGGTGGGC
Ribosomal protein L22	*RPL22*	6146	fw TGATTGCACCCACCCTGTAG rev GGTTCCCAGCTTTTCCGTTC
RUNX family transcription factor 2	*RUNX2*	860	fw: CCCACGAATGCACTATCC rev: GGACATACCGAGGGACA
Secreted phosphoprotein 1	*SSP1* (alias *OSP*)	6696	fw TGATTTTCCCACGGACCTGC rev TCGCTTTCCATGTGTGAGGT
TATA box binding protein	*TBP*	6908	fw CGGCTGTTTAACTTCGCTTCC rev TGGGTTATCTTCACACGCCAAG
TNF receptor superfamily member 11b	*TNFRSF11B* (alias *OPG*)	4982	fw: GAAGGGCGCTACCTTGA rev: GCAAACTGTATTTCGCTC
TNF superfamily member 11	*TNFSF11* (alias *RANKL*)	8600	fw: ATCACAGCACATCAGAGCAGA rev: TCACTTTATGGGAACCAGATGGG

## Data Availability

The datasets used and/or analyzed during the current study are available from the corresponding author upon reasonable request.
